# Electricity in the Diseases of Women

**Published:** 1890-07

**Authors:** 


ELECTRICITY IN THE DISEASES OF WOMEN, with special reference to the application of strong currents. By G. Betton Massey, M. D. Second edition, revised and enlarged. F. A. Davis, Publisher, 1231 Filbert Street, Philadelphia, 1890. Price, $1.50 net.

Less than a year ago, the first edition of this clever book was reviewed in these pages—August number, 1889, page 358. What was then said in praise of the book is equally applicable now that it has been enlarged and improved. The illustrations are excellent and of the latest types of electrical apparatus. One of the points about the book that catches our eye is the "Graphic Representation of the Law of Ohm," at page 219. It is excellent for all who are not mathematicians. There is an evident misprint in the last line at page 221, where the word *ampere* should be substituted for *Ohm*. W. M. J.

